# An analysis of movement patterns in mass casualty incident simulations

**DOI:** 10.1186/s41077-020-00147-9

**Published:** 2020-10-09

**Authors:** Boris Tolg, Juergen Lorenz

**Affiliations:** Hamburg University of Applied Sciences, Hamburg, Ulmenliet 20, Hamburg, 21033 Germany

**Keywords:** Mass casualty incident, Simulation, Movement patterns, Group recognition

## Abstract

**Background:**

Mass casualty incidents (MCI) such as train or bus crashes, explosions, collapses of buildings, or terrorist attacks result in rescue teams facing many victims and in huge challenges for hospitals. Simulations are performed to optimize preparedness for MCI. To maximize the benefits of MCI simulations, it is important to collect large amounts of information. However, a clear concept and standardization of a data-driven post-exercise evaluation and debriefing are currently lacking.

**Methods:**

GPS data loggers were used to track the trajectories of patients, medics, and paramedics in two simulated MCI scenarios using real human actors. The distribution of patients over the treatment area and their time of arrival at the hospital were estimated to provide information on the quality of triage and for debriefing purposes.

**Results:**

The results show the order in which patients have been treated and the time for the individual arrivals as an indicator for the triage performance. The distribution of patients at the accident area suggested initial confusion and unclear orders for the placement of patients with different grades of injury that can be used for post-exercise debriefing. The dynamics of movement directions allowed to detect group behavior during different phases of the MCI.

**Conclusions:**

Results indicate that GPS data loggers can be used to collect precise information about the trajectories of patients and rescue teams at an MCI simulation without interfering with the realism of the simulation. The exact sequence of the deliverance of patients of different triage categories to their appropriate destinations can be used to evaluate team performance for post-exercise debriefing. Future MCI simulations are planned to validate the use of GPS loggers by providing “hot-debrief” immediately after the MCI simulation and to explore ways in which group detection can provide relevant information for post-exercise evaluations

**Trial registration:**

Not applicable.

## Background

Mass casualty incidents (MCI) are situations in which the medical capacity of a region is insufficient to handle the volume and complexity of tasks [1]. MCI simulations are performed to optimize the preparedness of pre- and in-hospital care teams for MCI. However, MCI simulations are costly and complex. They require a lot of equipment and personnel, including actors coached to act out the behavior of victims and specially trained teams to provide them with make-up for realistic injury appearance. To guarantee the optimal benefit of MCI simulations, it is important to collect large amounts of information and data without interfering with the realism of the simulation. One major aim of MCI simulation is training the pre-hospital emergency care teams to perform the correct triage, i.e., organizing the sequence of delivery of patients to their appropriate destinations according to the severity of their injuries. Observation of these events is usually performed by passive observers in real time or by using video cameras at the various operational sites [2]. However, these approaches have limitations. The focus of the observers might be subjective, and video observation might miss important details. Additionally, it is important to maintain an optimal degree of immersion in the simulated scenario by a minimum of observers and visible measuring devices at the scene [3]. To analyze these complex situations, an objective measurement method is therefore required [4].

In recent years, radio-frequency identification (RFID) has been used in simulations to analyze the status of the injured simulation patients (SP) [5]. The collected data was compared to manual data collections to evaluate the reliability of the system. Proximity sensors have been used to gather information on interactions between individuals [6]. Although these technologies can be successfully applied in buildings, they have limitations in large and remote outdoor areas.

In this work, GPS data loggers were used to track movements of SP and participating medics and paramedics. A set of parameters was identified, to evaluate the actions of the rescue teams. The MCI participants are described as moving point objects (MPO). Every MPO has a unique id and at every time frame *t* a location *x*, *y*, *z*. MPOs have become an accepted norm in many publications in recent years [7, 8]. Movement data have been investigated by various approaches. Laube et al. described a relative motion (REMO) approach in which the data is transformed in a 2.5 dimensional matrix structure which is used to search and find basic motion patterns [9]. Dodge, Weibel und Lautenschütz describe a generic hierarchical framework for the classification of movement patterns [10]. In this article, we analyze the behavior of known groups as a foundation for future performance parameters.

Debriefing is an established practice used in medicine to facilitate reflexive learning as a fundamental element of an experiential learning process, both during simulation scenarios and in real working environments ([11, 12], for review, see [13]). Progress both in demonstrating the scientific evidence of its usefulness and in defining the skill demands on the debriefing instructor have predominantly been addressed in critical incident scenarios of small groups of participants, such as pre-hospital emergency or surgery teams with single SP, or in computerized patient simulators. There remains a significant lack of information as to how debriefing can be integrated and standardized in complex scenarios of MCI with different teams operating in separate locations. A particular challenge for MCI simulations is immediate post-exercise debriefing, referred to as ”hot debrief,” by which participants benefit from the fresh recall of events, cognitions, decisions, and emotions during the simulation. We examined, if the analysis of movement patterns provides information that may be used for a data-driven post-scenario debriefing of MCI simulations and develop a novel process of collecting the required data.

## Methods

The data for analysis was collected from two MCI training sessions performed in the same city (Essen, Germany) and by the same fire department in 2018 and 2019.

The first scenario was a van crashing into a group of people roaming in the woods next to a university hospital. The incident caused a total of *n*_*i*1_=18 persons to be injured. Simulation patients (SP) were brought to the nearby (≈300*m*) hospital, which was involved in the training. A total of *N*_1_=127 persons were involved in the simulation. The scenario was stopped, before all patients were brought to the hospital. For this reason, the data of the first scenario is incomplete.

The movements of the SPs and the rescuers were tracked with *M*_1_=44 GPS-loggers. Table 1 shows the distribution of the GPS-loggers to the participants of the simulation. It was possible to track the movement of all SPs. Unfortunately, the number of trackers was not sufficient to track all rescuers, so it was decided to track the movement of two of the rescue teams, which were working cooperatively.

**Table 1:** Distribution of GPS-loggers to participants of study 1

**Number of GPS-loggers**	**Tracked group**
*m*_*r*1_=8	Patients with category red
*m*_*y*1_=5	Patients with category yellow
*m*_*g*1_=5	Patients with category green
*m*_*f*11_=7	Rescue team 1
*m*_*f*21_=8	Rescue team 2
*m*_*o*1_=2	Organizational leaders
*m*_*l*1_=1	Leading emergency doctor
*m*_*x*1_=8	Not used

The second scenario was a fire and an explosion in a complex cellar environment. In this case, *n*_*i*2_=23 persons were injured. The hospital was approximately 4*km* away and the transport of the patients was realized with several ambulances and one helicopter. The hospital was part of the training. In this simulation *N*_2_=156 persons were involved.

In the second scenario, the movement of the participants was tracked with *M*_2_=123 GPS-loggers. Table 2 shows the distribution of the GPS-loggers to the participants.

**Table 2:** Distribution of GPS-loggers to participants of study 2

**Number of GPS-loggers**	**Tracked group**
*m*_*r*2_=8	SPs category red
*m*_*y*2_=4	SPs category yellow
*m*_*g*2_=11	SPs category green
*m*_*f*12_=8	Rescue team 1
*m*_*f*22_=6	Rescue team 2
*m*_*f*32_=4	Rescue team 3
*m*_*a*12_=2	Ambulance 1
*m*_*a*22_=3	Ambulance 2
*m*_*a*32_=2	Ambulance 3
*m*_*a*42_=3	Ambulance 4
*m*_*a*52_=4	Ambulance 5
*m*_*a*62_=1	Ambulance 6
*m*_*a*72_=2	Ambulance 7
*m*_*a*82_=3	Ambulance 8
*m*_*a*92_=2	Ambulance 9
*m*_*a*102_=2	Ambulance 10
*m*_*a*112_=2	Ambulance 11
*m*_*o*2_=2	Organizational leaders
*m*_*l*2_=1	Leading emergency doctor
*m*_*x*2_=43	Not used

All patients were grouped in three categories defined by the mSTaRT[14] triage system. The SPs were represented by trained actors who got medical instructions about their injuries. The treatment and transport of the injured was undertaken by various rescue teams which established a staging area for the ambulances as well as a treatment area for the SPs and a rest area for those without injuries.

All GPS-loggers were equipped with a unique identification number (ID). Participants had to answer a questionnaire which is not part of this report including their logger ID, their affiliation and their role in the training. This included the case number identifying the planned injuries for SPs. No personal data was gathered.

GPS loggers were distributed to the rescue teams and the SPs before the start of the simulation. They were hidden beneath clothes or in pockets to minimize any influence on the realism of the simulation. The GPS data loggers were collected after the training.

### Algorithm to estimate the time to hospital

An obvious parameter that allows an evaluation of the efficiency of the participants in a training scenario is the time taken to organize transport of patients to hospital. This parameter was realized by defining a destination area *DA*_*g*_,*DA*_*y*_,*DA*_*r*_ for the green, yellow, and red categorized SPs. The time measurement started with the emergency call at the beginning of the training. The duration of the training was subdivided into 10*s* frames *t* with *t*∈{0,…,*T*−1}. Let *F*_*t*_(*o*) be the position of participant *o* at the time frame *t*, known as the ”fix” of *o* at time frame *t* represented by a vector $\left (\begin {array}{c}x\\y\end {array}\right)$on a 2D plane.

Let *g*(*DA*,*t*,*o*) be a function indicating whether an object *o* is within a destination area *DA* at a given time frame *t* with

1 $$\begin{array}{@{}rcl@{}} g(DA,t,o) = \left\{\begin{array}{ll} 1,&\text{when}\ {F}_{t}(o)\ \text{is within}\ {DA}\\ 0,&\text{otherwise} \end{array}\right. \end{array} $$

Then, the percentage of participants *o* of a Group *O* at a time frame *t* is called *G*(*DA*,*t*,*O*) and is defined by

2 $$\begin{array}{@{}rcl@{}} G(DA,t,O) = \left\{\begin{array}{ll} \frac{1}{m_{o}}\sum_{o\in O}{g(DA,t,o)} \end{array}\right. \end{array} $$

with *m*_*O*_ being the number of participants in group *O*. In a training simulation, the behavior of the SPs differs depending on the scenario. In both training sessions, the SPs left the simulation when they arrived at the hospital. They subsequently roamed around, either observing of the training session or leaving the area. For the remaining simulation period, the movement data of those SPs was recorded as their last position at the hospital.

To analyze the differences between the arrival times of the categories the Wilcoxon test was performed between all pairs.

### Algorithm to evaluate the regions of interest

For the training analysis, it is important to consider where the patients are assembled and treated. Were the different triage categories assembled at the same place, or were they mixed? Were the green patients separated from the others to minimize interferences?

To analyze the regions of interest of the groups, we created heatmaps based on a 2D histogram of the movement of group members. The area was subdivided into squares of roughly 9*m*^2^. Let *A*_*ij*_ be the square at position (*i*,*j*), with *i*∈{0,…,*I*−1} and *j*∈{0,…,*J*−1}. Let *h*(*i*,*j*,*t*,*o*) be a function indicating whether an Object *o* is within a square *A*_*ij*_ at a given time frame *t* with

3 $$\begin{array}{@{}rcl@{}} h(i,j,t,o) = \left\{\begin{array}{ll} 1,&\text{when}\ {F}_{t}(o)\ \text{is within}\ {A}_{{ij}}\\ 0,&\text{otherwise} \end{array}\right. \end{array} $$

Then, the 2D histogram *H*(*i*,*j*,*o*) is defined by

4 $$\begin{array}{@{}rcl@{}} H(i,j,o) = \left\{\begin{array}{ll} \sum_{t=0}^{T-1}{h(i,j,t,o)} \end{array}\right. \end{array} $$

The regions of interest are generated as an accumulation of all movements during the study.

### Parallel movement analysis to detect group behavior

Before the analysis started, all movement patterns have been manually assigned to groups. The groups of both studies were listed in Tables 1 and 2.

To detect group behavior, the angle between the directions of motion of each pair of MPOs is measured. This parameter is insensitive to the actual direction of motion and describes only the relative motion between two MPOs. If the angle is near zero, both MPOs walk in approximately the same direction. If the angle is near *π* the movement is opposing.

The direction of motion *D*_*t*_(*o*) of an object *o* at the time frame *t* can be defined by *F*_*t*+1_(*o*)−*F*_*t*_(*o*). The normalized direction shall be $\hat {D_{t}(o)}$. Then, the angle *A*_*t*_(*o*_1_,*o*_2_) between the direction of two objects *o*_1_ and *o*_2_ can be calculated with the vector dot product:

5 $$\begin{array}{@{}rcl@{}} A_{t}(o_{1},o_{2}) = \cos^{-1}\left(\hat{D_{t}(o_{1})}\cdot\hat{D_{t}(o_{2})}\right) \end{array} $$

The value of *A*_*t*_(*o*_1_,*o*_2_) is in the interval [0,*π*].

To compare two groups, we define two sets for every time frame *t*. The first set contains the angles of the directions of motion only between members of the first group. The second set contains the angles between members of the first group and members of the second group.

Let us assume that there are *Θ* groups within the training. To compare the movement of the members of one group *O*_*α*_ with the movement of the members of the other groups, two sets *Γ* and *Ψ* are created, with

6 $$ {}\Gamma_{{ta}} = \left\{ A_{t}(o_{\iota}, o_{\kappa}) \vert\iota < \kappa, o_{\iota}, o_{\kappa} \in O_{\alpha} \right\}  $$

7 $$ {}\Psi_{{ta}}= \left\{ A_{t}(o_{\iota}, o_{\kappa}) \vert o_{\iota} \in O_{\alpha}, o_{\kappa} \in O_{\delta},\alpha \neq \delta,\forall \delta \in \left\{ 0,\ldots, \Theta\right\}\right\}  $$

Since the distribution of both sets cannot be assumed as normal, and the size of the sets can be small, the Wilcoxon test was performed on both groups to identify significant differences in behavior.

## Results

### GPS-based estimation of time to hospital

For both studies the time was estimated to deliver SPs of the three categories (red, yellow, and green) to the hospital. The percentage of group members within the destination area was calculated using Eq. 2. The time measurement started with the emergency call at the beginning of the training.

**Fig. 1:**
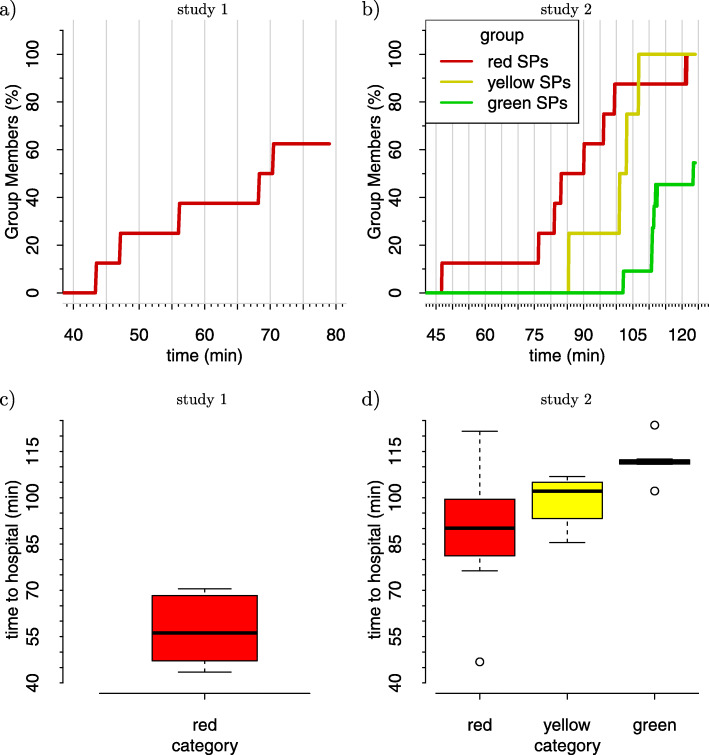
Time sequences for the time to hospital. **a** Only category red in study 1. **b** All three categories in study 2. Boxplot representation **c** for study 1 and **d** for study 2

Figure 1a shows the estimated time for category red SPs in study 1 be delivered to hospital. SPs in other categories were not delivered to the hospital in this training session. It took about 70 min to deliver 60% of the category red SPs to the hospital. Figure 1b shows the same measurement for all three categories in the second study. The three categories had different priorities which can be seen in the order of arrivals at the hospital.

The boxplot of the time to hospital for the SPs in study 2 is shown in Fig. 1d. The first SP of the red category was delivered to the hospital 47 min after the start of the measurement. The median time for the category red was about 90 min. The delivery of SPs of the category yellow started at 85 min with a median of 100 min. The delivery of green SPs was not complete, but started 102 min after the start of the measurement with a median value of 110 min.

The differences between the delivery times of the different categories were analyzed using the Wilcoxon test. While the difference between the red and the yellow category was not significant (*p*=0.33), both the difference between the red and green category (*p*=0.04) and the yellow and green category (*p*=0.02) were significant.

Although a direct comparison is not possible, the boxplot of the time to hospital for the red SPs in study 1 is shown in Fig. 1c. The duration to deliver 60% of the red SPs was only 27 min in study 1 (measured from the first delivered SP) instead of 43 min in study 2, but the categories yellow and green were ignored.

The differences in the delivery times between study 1 and 2 can be explained by the location of the training. In study 1, the training was located within a radius of 300*m* around the hospital with few traffic. The accident in study 2 was within a 4*km* radius around the hospital and the ambulances had to drive through the city. A planning software calculated 7 min for the route.

### Region of interest analysis of the operational area

**Fig. 2:**
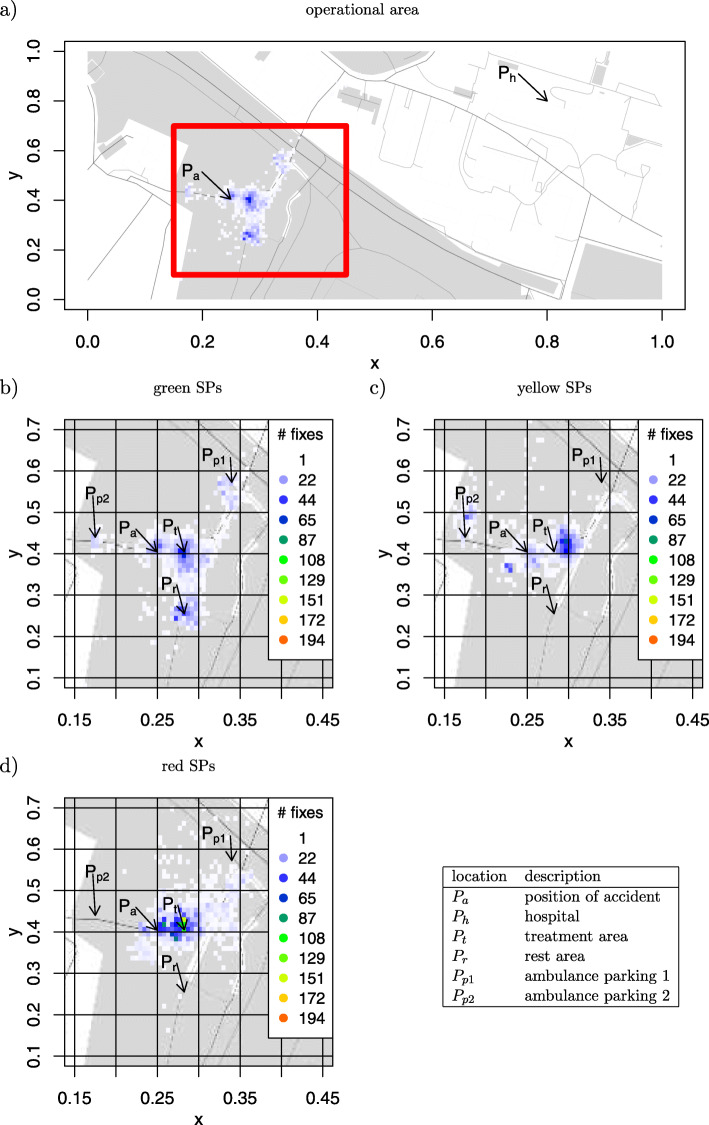
2D histogram for movement patterns in study 1. **a** Display of the complete training area. **b** Magnification of the movements of green SPs within the treatment area. **c**) Magnification of the movements of yellow SPs within the treatment area. **d** Magnification of the movements of red SPs within the treatment area

**Fig. 3:**
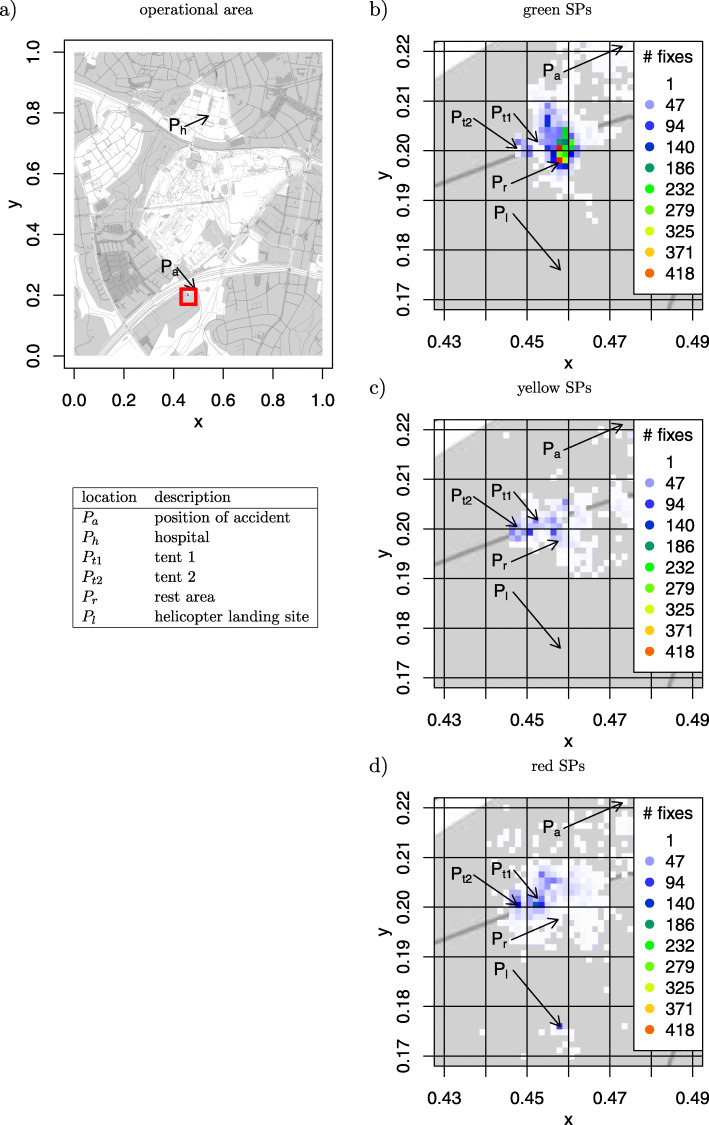
2D histogram for movement patterns in study 2. **a** Display of the complete training area. **b** Magnification of the movements of green SPs within the treatment area. **c** Magnification of the movements of yellow SPs within the treatment area d) magnification of the movements of red SPs within the treatment area

The full operational area is shown in Fig. 2a for the first study and in Fig. 3a for the second study. Since simulations of the MCI trainings are predominantly located at the treatment area *P*_*t*_, Fig. 2b to d show magnifications of this location for study 1, while Fig. 3b to d show magnifications for the second study.

The regions of interest defined by Eq. 4 were calculated for the three SP categories green, yellow, and red. The heatmap for the green SPs show five regions of interest (ROI) in the treatment area (Fig. 2b).

For study 1, *P*_*a*_ (Fig. 2b) marks the position of the accident. The same location is highlighted for yellow and red SPs in Fig. 2c and d. Simulation patients were treated at *P*_*t*_ which are highlighted in all three figures. Red SPs have been delivered to the hospital by the ambulances at *P*_*p*1_ and yellow SPs by the ambulances at *P*_*p*2_. The green SPs followed some of the other SPs to the ambulances. Green SPs were separated from the others to give them some rest and to minimize interruptions of minimally injured SPs who do not need immediate treatment. The ROI at the coordinates *P*_*r*_ shows where the green SPs had to wait.

The ROI at *P*_*p*2_ for yellow SPs (Fig. 2c) locates the pickup point for the ambulances bringing the yellow SPs to the hospital. This point is hot on the plot because the SPs were not actually delivered, but waited in the ambulance. Red SPs were brought to the ambulance at *P*_*p*1_ which immediately left for the hospital causing only a few marks on the heatmap (Fig. 2d).

The results show that all SPs were located most of the time around the treatment area *P*_*t*_. The green SPs can be found at all relevant regions, since they followed other patients to the ambulance and did not stay at the rest area *P*_*r*_.

The operational area of the second study (Fig. 3a) was larger than in the first study, since the hospital was about 4*km* away from the accident. In this scenario, the ambulances had to drive through the city to deliver SPs to the hospital. This causes time intervals for the ambulances to return to the training area.

In this scenario, the SPs were placed in a cellar room, so that no GPS fixes could be generated over that time period. The heatmaps will therefore show only a few fixes at the area of the accident *P*_*a*_.

Following classification, green SPs were separated from the other patients to minimize interruption. Their main activities are located around *P*_*r*_ slightly further east of the other categories (Fig. 3b).

There are two ROIs within that heatmap for yellow SPs at *P*_*t*1_ and *P*_*t*2_ (Fig. 3c). In this second study, the rescue teams used two tents to establish treatment areas for yellow and red SPs. The yellow SPs were scattered in both tents at the beginning and gathered in *P*_*t*2_ after some time. The two ROIs indicate the scattering of yellow SPs during the execution of the training session. Red SPs are mostly located at *P*_*t*_1 but also at *P*_*t*2_.

The heatmaps show that the green SPs stayed at *P*_*r*_ for some time indicating that their priority was lower.

One red SPs was delivered to the hospital with a helicopter at *P*_*l*_. Since it was only one particular SP, we know that all fixes were caused by only one GPS logger. Thus, we know that the red SP stayed at *P*_*l*_ for at least 94/6≈15 min. This is an artificial effect of the training, since the helicopter could not fly to the hospital with high priority.

We discussed our results with the involved leaders of the fire department after both training sessions. All regions of interest were confirmed by their documentation. The manually recorded times for the delivery of the patients to the hospital were incomplete. The categories assigned to the SPs have to be documented for further research to allow conclusions about the decisions made during the training sessions.

### Analysis of the parallel movement for group identification

**Fig. 4:**
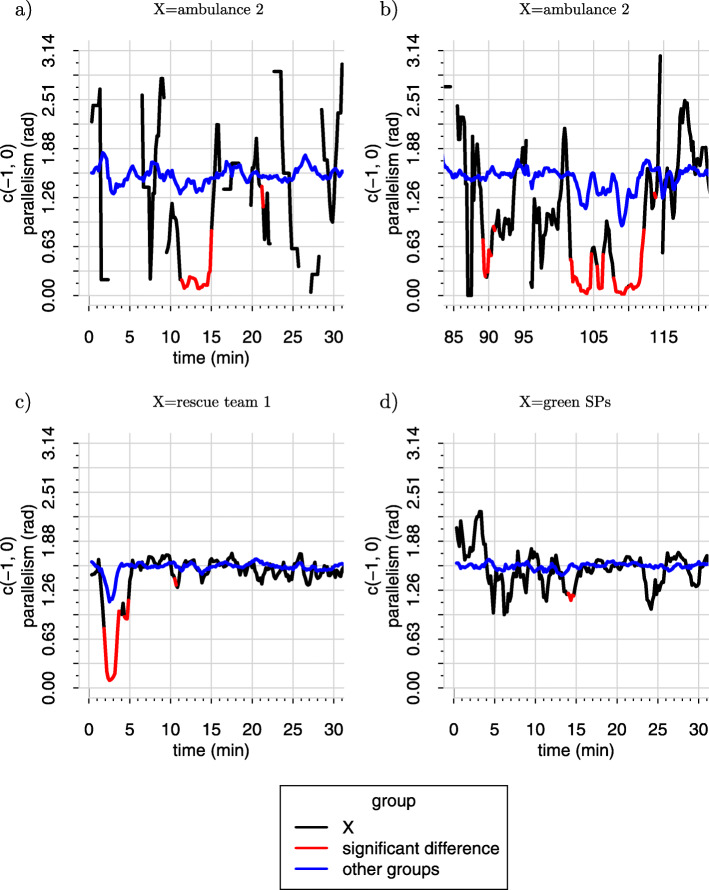
Parallel movement in study 2. **a**, **b** Between members of the ambulance 2.**c** Between members of fire brigade 1. **d** Between members of the green SPs. The parallel movement is compared to all members of other groups

The analysis of parallel movement is shown in Fig. 4 for both studies. These results are not intended to be used in debriefing but as a first step for a behavioral analysis.

The black lines always represent the mean of all members of *Γ*_*ta*_, which is the mean parallel movement within the group under consideration for every time frame *t*. To smooth the representation, we used a two-sided moving average with a 1-min window. The blue line represents the mean of all members of *Ψ*_*ta*_ which is the mean parallel movement of every group member compared to any member of any other group. The Wilcoxon test was used to compare both sets. If the Wilcoxon test shows a significant *p* value below 0.05, the black line was marked red for that time frame *t*.

Figure 4a and b show the analysis of the movement vectors of the members of ambulance 2 in the second study. The analysis of ambulances is difficult, since they contain only two to four members. In many cases, one or both crew members did not move; hence, it was impossible to calculate the dot product within the members of the group or to members of other groups. Also the Wilcoxon test could frequently not be applied, since the group was too small.

The movement of the subjects in the ambulances is aligned for values between 0 and 0.35, when they are moving. This represents their behavior in an MCI training. They are either waiting for SPs or delivering them to the hospital. In the case of ambulance 2, the Wilcoxon test showed several significant differences in behavior from members of other groups. We will call a group with significantly aligned behavior a *behavioral group*.

In Fig. 4c, the movement of the subjects in rescue team 1 shows an aligned behavior from minutes 1 to 4 and from minutes 4 to 5. The first aligned movement represents the arrival of the rescue team at the site of the accident with their truck. The second is the collective movement from the truck to the accident. For at least 1 min, both parallel movements are significantly different from the movement of the members of all other groups.

An aligned movement in the minutes around the arrival at the site of the accident that is significantly different to the movement of all members of all other groups can be seen for all members of fire departments in both trainings.

There are sometimes other peaks, which indicate aligned behavior, but not all of them are significant and none of them lasts as long as 1 min. The members of rescue teams show the characteristics of a behavioral group.

In both training sessions, the approach of the rescue teams and ambulances was extremely short, since all vehicles started relatively close to the site of the accident. It can be assumed that the aligned behavior would last longer in case of a real event.

Figure 4d shows the parallel movement for the SP category green in study 1. This behavior was comparable for all SPs in both trainings. There are sometimes peaks, which are significantly different to the behavior of all other group members but none of them lasts for a minute. In most cases the behavior shows no significant difference.

This might indicate that the SPs form a different kind of group than the rescue teams or ambulances. While the rescue teams have a common command structure and a common goal, the SPs do not. We will call a group with common traits but without common behavior a *trait group*.

To counteract the problem of multiple comparisons we applied the Bonferroni correction to our results. The significant segments in Fig. 4a and b vanished but the significant segments in Fig. 4c and d were still significant.

## Discussion

Our study aimed to use GPS loggers to collect information about the cumulative presence times and movements of participating simulation patients (SPs) and rescue teams to evaluate the time duration of the triage in two mass casualty incident simulations (MCI). Within the MCIs, an operational area was defined by several sub-locations, such as the accident site, the treatment area for severely injured (category red and yellow), and rest areas in which less severely injured SPs (green) were separated and taken care of according to their individual needs of subordinate priority.

The GPS loggers of the SPs perfectly allowed to visualize the successful separation of casualties according to their triage category within these sub-locations at the operational area. It especially demonstrated that category green SPs were separated from the others to reduce interferences. The distribution of yellow and red SPs in the second training session suggested initial confusion and unclear orders for the placement of patients. Furthermore, the GPS loggers provided data to estimate the time from the start of the simulation to the delivery at the hospital that yielded significant differences according to the triage category with red SPs delivered in first, yellow in second, and green in third place. There was no indication that the GPS loggers were influencing the flow of actions or disturbed the realism of the simulation.

Recently, Ozella et al. demonstrated the use of wearable proximity sensors in MCI simulation of a simulated collapse of a building to track the flow of patients from the accident site to the hospital that allowed to quantitatively record the contact times between nurses and doctors with the victims according to their triage category [6]. Our results agree with their conclusion that technologies such as GPS loggers or wearable proximity sensory are non-intrusive in terms of maintaining optimal immersion and can be used to facilitate the post-exercise debriefing by which participants gather relevant information about successes and deficiencies to improve the preparedness for MCI in future trainings or real events.

Furthermore, we tested the use of data that allowed to describe parallelism of movements. It demonstrated group identities during particular phases of the simulation, such as during arrival of rescue teams or their evacuation of casualties. In between these events, such as during triage and treatment phases, different groups ”diffused” into each other. Currently, we continue to explore the utility of these information for post-exercise debriefing in how group behavior demonstrates effectiveness of rescue teams during an MCI simulation. Kash et al. stated the necessity of tools to measure team effectiveness which are critical to improve health outcomes and can help to increase health care quality [15]. Although our results do not allow such conclusions yet, the development of group performance measures based on GPS logger data might be the first step.

Our current approach has a number of limitations. It is limited to outdoor scenarios and GPS logs need to be manually transferred to the database. Currently, this process takes several hours, which strongly reduces the use of GPS logs for an immediate post exercise debriefing (”hot debrief”). Future applications are planned in which the destination areas are already modeled during the planning of the simulation. We are also developing a smartphone app, which collects the anonymous information of the participant. The app sends location data live to the database and combines GPS, WiFi, and Bluetooth data for both outdoor and indoor positioning. The app allows direct live monitoring of the events on screen and the results are available minutes after the simulation. We are improving the stability of the app and first tests show that the results can be used for a hot debriefing which is a standard in simulation based learning scenarios, e.g., used in settings for crisis resource management (CRM) trainings[16]; for review, see [17].

## Conclusions

The results show that the duration of stay and movement data collected from GPS loggers can be used to analyze and evaluate MCI trainings. The time can be estimated for different triage categories of casualties. The region of interest visualized in heatmaps of cumulative durations of stay allow the evaluation of the triage performance. The parallel movement analysis allows to detect groups that share common behavior, like the approach to and evacuation from the accident site of rescue teams.

Future studies may be designed with a great variety of specific aims, such as addressing questions of how much human resources or technical equipment are needed to handle a given MCI. Here, effective evaluation tools can help to standardize quality assessment of public preparedness for mass casualty incidents.

## Data Availability

The datasets used and/or analyzed during the current study are available from the corresponding author on reasonable request.

## Abbreviations

EMS: Emergency medical service
ROI: Region of interest
MCI: Mass casualty incident
SP: Simulation patient

## References

[1] Admi H, Eilon Y, Hyams G, Utitz L (2011). Management of mass casualty events: the israeli experience. J Nurs Sch Off Publ Sigma Theta Tau Int Honor Soc Nurs Sigma Theta Tau.

[2] Kaji A, Langford V, Lewis R (2008). Assessing hospital disaster preparedness: a comparison of an on-site survey, directly observed drill performance, and video analysis of teamwork. Ann Emerg Med.

[3] Ingrassia PL, Carenzo L, Barra FL, Colombo D, Ragazzoni L, Tengattini M, Prato F, Geddo A, Della Corte F (2011). Data collection in a live mass casualty incident simulation: automated RFID technology versus manually recorded system. Eur J Emerg Med Off J Eur Soc Emerg Med.

[4] Ingrassia PL, Prato F, Geddo A, Colombo D, Tengattini M, Calligaro S, La Mura F, Franc J, Della Corte F (2009). Evaluation of medical management during a mass casualty incident exercise: an objective assessment tool to enhance direct observation. J Emerg Med.

[5] Ganz A, Schafer J, Tang J, Yang Z, Yi J, Ciottone G (2015). Urban search and rescue situational awareness using DIORAMA disaster management system. Procedia Eng.

[6] Ozella L, Gauvin L, Carenzo L, Quaggiotto M, Ingrassia PL, Tizzoni M, Panisson A, Colombo D, Sapienza A, Kalimeri K, Della Corte F, Cattuto C. Wearable proximity sensors for monitoring a mass casualty incident exercise: feasibility study. J Med Int Res. 2019; 21. 10.2196/12251. 10.2196/12251 PMC6658323 31025944

[7] Iwase S, Saito H. Tracking soccer players based on homography among multiple views: 2003. p. 283–92. 10.1117/12.502967.

[8] Grönroos S, Peltonen L-M, Soloviev V, Lilius J, Salanterä S (2017). Indoor positioning system for movement path analysis in healthcare institutions. Finn J eHealth eWelfare.

[9] Laube P, Kreveld MJ, Imfeld S. Finding remo - detecting relative motion patterns in geospatial lifelines. Dev Spat Data Handl. 2005. 10.1109/ares.2012.68.

[10] Dodge S, Weibel R, Lautenschütz AK (2008). Towards a taxonomy of movement patterns. Inf Vis.

[11] Dieckmann P, Molin S, Lippert A, Østergaard D (2009). The art and science of debriefing in simulation: ideal and practice. Med Teach.

[12] Oriot D, Alinier G. Pocket book for simulation debriefing in healthcare; 2018. 10.1007/978-3-319-59882-6.

[13] Fanning R, Gaba D (2007). The role of debriefing in simulation-based learning. Simul Healthc J Soc Simul Healthc.

[14] Benson M, Koenig K, Schultz C (1996). Disaster triage: start, then save-a new method of dynamic triage for victims of a catastrophic earthquake. Prehospital Disaster Med.

[15] Kash B, Cheon O, Halzack N, Miller T (2018). Measuring team effectiveness in the health care setting: an inventory of survey tools. Health Serv Insights.

[16] Rall M, Manser T, Howard S (2000). Key elements of debriefing for simulator training. Eur J Anaesthesiol.

[17] Rall M, Dieckmann P (2005). Simulation and patient safety: the use of simulation to enhance patient safety on a systems level. Curr Anaesth Crit Care.

